# Reconciling Local Structure Disorder and the Relaxor State in (Bi_1/2_Na_1/2_)TiO_3_-BaTiO_3_

**DOI:** 10.1038/srep31739

**Published:** 2016-08-22

**Authors:** Pedro B. Groszewicz, Melanie Gröting, Hergen Breitzke, Wook Jo, Karsten Albe, Gerd Buntkowsky, Jürgen Rödel

**Affiliations:** 1 Institute of Physical Chemistry, Technische Universität Darmstadt, 64287, Darmstadt, Germany; 2 Institute of Materials Science, Technische Universität Darmstadt, 64287, Darmstadt, Germany; 3 School of Materials Science and Engineering, Ulsan National Institute of Science and Technology, 689-798, Ulsan, Republic of Korea

## Abstract

Lead-based relaxor ferroelectrics are key functional materials indispensable for the production of multilayer ceramic capacitors and piezoelectric transducers. Currently there are strong efforts to develop novel environmentally benign lead-free relaxor materials. The structural origins of the relaxor state and the role of composition modifications in these lead-free materials are still not well understood. In the present contribution, the solid-solution (100-*x*)(Bi_1/2_Na_1/2_)TiO_3_-*x*BaTiO_3_ (BNT-*x*BT), a prototypic lead-free relaxor is studied by the combination of solid-state nuclear magnetic resonance (NMR) spectroscopy, dielectric measurements and *ab-initio* density functional theory (DFT). For the first time it is shown that the peculiar composition dependence of the EFG distribution width (ΔQIS_width_) correlates strongly to the dispersion in dielectric permittivity, a fingerprint of the relaxor state. Significant disorder is found in the local structure of BNT-xBT, as indicated by the analysis of the electric field gradient (EFG) in ^23^Na 3QMAS NMR spectra. Aided by DFT calculations, this disorder is attributed to a continuous unimodal distribution of octahedral tilting. These results contrast strongly to the previously proposed coexistence of two octahedral tilt systems in BNT-xBT. Based on these results, we propose that considerable octahedral tilt disorder may be a general feature of these oxides and essential for their relaxor properties.

Owing to the environmental hazards of lead there are currently intense world-wide research efforts to develop novel lead-free substitution materials for electric and electronic applications. Bismuth-based piezoelectric perovskites based on solid-solutions of (Bi_1/2_Na_1/2_)TiO_3_ (BNT) and BaTiO_3_(BT) have emerged as one of the most promising classes of environment friendly substitutes for lead-based ceramics^1^,^2^,^3^. The piezoelectric properties of (100-*x*)(Bi_1/2_Na_1/2_)TiO_3_-*x*BaTiO_3_ (BNT-*x*BT, 0 ≤ x ≤ 15) are optimal at a barium content around 6–7%^4^,^5^.

Depending on the barium content (xBT)^6^, the BNT-xBT solid-solutions are found either in a relaxor state or in a ferroelectric state. In the relaxor state the electric polarization is highly localized and displays short-range coherence only, in the form of nanodomains^2^,^6^. The ferroelectric state is characterized by a long-range ordering of electric dipoles. By electric poling, the relaxor state can be switched to the ferroelectric state^5^, which can be converted back to the relaxor state by thermal annealing at elevated temperatures.

The addition of BT not only allows one to tune the electric state of these solid-solutions, but it also results in a series of composition-induced phase transitions. The parent BNT exhibits *R3c* rhombohedral global symmetry^7^, which is also stable at small barium contents, like for example BNT-4BT^6^. With further addition of BT, the symmetry changes to tetragonal^4^, either with octahedral tilting (P*4bm*) for a barium content 6 < x < 10 or without tilting (P*4mm*) for compositions with x > 11^6^.

However, the structure of BNT-*x*BT, together with its relation to macroscopic properties and the electric state, remain controversial issues. For pure BNT, for example, the *R3c* symmetry was first assigned to its room-temperature structure^7^, while high-resolution X-ray diffraction indicates a monoclinic *Cc* structure with displacements of bismuth cations away from the [111] direction^8^. The assignment of C*c* symmetry to the global structure has been debated in an extensive diffraction study^9^, but further supported by a recent TEM investigation^10^, illustrating the lack of consensus in this matter.

A second disputed point regards the apparently cubic symmetry of compositions with intermediate barium content, well-exemplified by BNT-6BT. Although first indications from X-ray diffraction suggested that the average structure is cubic^11^, TEM experiments revealed the concurrent presence of tetragonal and rhombohedral distortions, manifested in the presence of the respective superstructure reflections^5^,^12^. More recently, an NMR study of the local structure of BNT-*x*BT demonstrated that a cubic phase coexists with a phase of lower symmetry in BNT-6BT^13^, as evidenced by the relative intensity of signals from different ^23^Na NMR transitions. Their shape also indicated a distribution of local environments, suggesting some kind of structural disorder in these A-site substituted ceramics.

The complexity of the structure of BNT can be attributed to very subtle changes in the octahedral tilt system, which corresponds to rotations of TiO_6_ octahedra and are conveniently described in the Glazer notation^14^. The original rhombohedral (*R3c*) structure reported for room temperature BNT exhibits an anti-phase (*a*^−^*a*^−^*a*^−^) tilt system^7^, whereas the high temperature tetragonal structure is described by an in-phase (*a*^*0*^*a*^*0*^*c*^+^) tilt system^7^. At a later point, a TEM investigation by Dorcet and Trolliard demonstrated the coexistence of *a*^−^*a*^−^*a*^−^ and *a*^*0*^*a*^*0*^*c*^+^ systems^12^. Besides that, an additional tilt system was suggested to evolve in pure BNT during heating, for which twin boundaries between *a*^−^*a*^−^*a*^−^ domains result in sheets of *Pnma* symmetry with *a*^−^*a*^−^*c*^+^ tilt^15^. Later, Levin and Reaney^16^ reported on a structure model that rationalizes the average monoclinic symmetry of BNT as a coexistence of in-phase and anti-phase tilting, the first exhibiting a significantly shorter coherence length than the latter. In addition, they proposed that the relative magnitude and direction of octahedral tilting may vary continuously in order to accommodate the strain associated with boundaries between the differently oriented tilted regions.

For the barium-modified compositions, coexistence between in-phase and anti-phase tilt systems was also observed in BNT-6BT by TEM^5^ and further supported by an XRD study^9^. Such phase coexistence, however, is not a feature of all BNT-*x*BT compositions. For example, it has been recently shown that BNT-3BT is characterized by the single tilt system *a*^−^*a*^−^*a*^−^10.

In summary, several characterization techniques indicate the coexistence of different octahedral tilt systems for BNT-xBT on a short length scale, which may be alternatively understood as tilt disorder. Furthermore, the degree of tilt disorder can be governed by the barium content of a given (100-x)BNT-xBT composition.

The diffraction and microscopy studies described above rely mostly on characterization of the average crystal structure. However, one can expect the local structure to be different from the average one in these solid solutions, due to the mixed occupation of the perovskite A-site by Bi^3+^, Na^+^ and Ba^2+^. Each specific arrangement of A-site cations may induce a structural distortion that leads to a local deviation from the average symmetry. This idea is supported by DFT calculations, which have demonstrated that different local chemical arrangements stabilize different tilt systems in BNT^17^. The random A-site occupancy, which was found in TEM studies (high-angle annular dark field)^16^,^18^ and diffuse X-ray scattering^19^, guarantees the presence of a wide variety of local cation arrangements in these chemically disordered perovskites.

NMR is an adequate tool for the characterization of the local structure in such disordered materials. Owing to the close range of interactions between the probed nucleus and its near surroundings^20^,^21^, NMR is a probe of the structure at the atomic scale. Furthermore, each local environment delivers a specific fingerprint, independent of the presence of long-range order. However, disordered features in the structure also pose challenges to NMR spectroscopy, as they result in a distribution of signals due to the various local environments around the studied nuclei. In addition to that, solid-state NMR spectra are usually the outcome of several overlapping interactions between the probed nucleus and its local environment (e.g. chemical shift and quadrupolar coupling), which further hinders the analysis of a single interaction. In the case of elements with quadrupolar nuclei, such as ^23^Na, the triple-quantum magic angle spinning (3QMAS) NMR experiment is able to overcome the latter issue^22^. The 3QMAS is a two-dimensional spectroscopic method able to effectively separate the contribution of the quadrupolar interaction from the chemical shift one. This feature provides higher resolution and allows the variety of local interactions to be described as a distribution of structure-related NMR parameters, as for instance the electric field gradient (EFG).

The electric field gradient on the site of a given nucleus is determined by the electric charge distribution in the surroundings, which is generated by its own electrons and the neighboring ions. This tensor quantity is especially sensitive to the immediate vicinity of the nucleus, as it scales with r^−3^^20^,^21^. A simply point-charge calculation shows that ions located in the nearest neighbor shell and the second nearest neighbors contribute to ca. 85% of the observed EFG. For the ideal perovskite structure with cubic symmetry, the EFG on the A-site is equal to zero. Any breaking of the cubic point symmetry at the A-site will cause an EFG≠0. The size of the EFG depends on the magnitude of the structural distortion that causes it. Three different mechanisms of structural distortion may be responsible for an EFG≠0 in the A-site of BNT-xBT: (1) an asymmetric arrangement of neighboring A-site cations due to dissimilar electric charges of Na^+^, Ba^2+^ and Bi^3+^; (2) polar displacements of all ions, due to the close relationship between electric polarization and the EFG^23^,^24^,^25^; (3) tilting of oxygen octahedra, which causes a deformation of the oxygen cuboctahedron around the A-site cation and results in an asymmetric charge distribution.

Despite the direct connection between the EFG and a material’s structure, the interpretation of NMR spectra in terms of structural properties of a material is not unique. For this reason structural modeling is necessary. In particular, *ab-initio* calculations can provide the link between the local structure of complex perovskites and NMR relevant parameters as the EFG. Since it has been demonstrated that projector augmented wave (PAW) based density functional theory (DFT) calculations yield reasonable values of electric field gradient components in solids^26^, several combined experimental-theoretical studies of perovskites have been published. There are, for example, DFT-assisted assignments of crystal structures based on electric field gradients for NaNbO_3_^27^,^28^,^29^, PbZr_1-*x*_Ti_*x*_O_3_^30^ or La_*x*_Y_1-*x*_ScO_3_^31^. Besides being a suitable method to assist the interpretation of NMR spectra, DFT calculations are also a well-suited tool to explore the relationship between electric field gradients and structural features in order to reveal general structure-property relations.

In this study, the local structure of (100-*x*)(Bi_1/2_Na_1/2_)TiO_3_-*x*BaTiO_3_ (BNT-*x*BT, 0 ≤ x ≤ 15) ceramics is investigated by means of ^23^Na nuclear magnetic resonance spectroscopy (NMR). The 3QMAS NMR experiment provides information about the local structure in terms of the electric field gradient (EFG) and its distribution. With aid of *ab-initio* density functional theory (DFT) calculations, three possible distortion mechanisms are evaluated with respect to the EFG magnitude and the role of the presence of barium in the structure. These results are interpreted in light of the electric state determined for each composition with dielectric spectroscopy, in order to reveal the role that local structural features play for the irregular occurrence of relaxor behavior in BNT-xBT.

## Results

**Figure 1:**
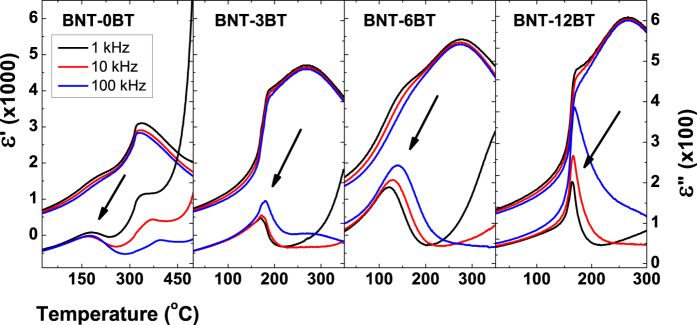
Real (ε’) and imaginary part (ε”) of the temperature-dependent dielectric permittivity of unpoled specimens for selected BNT-xBT compositions. Arrows indicate the influence of increasing frequency of the temperature of the maximum in ε” (ΔT_m_). Note the sharp inflexion point in ε’ is only present for BNT-3BT and BNT-12BT samples.

**Figure 2:**
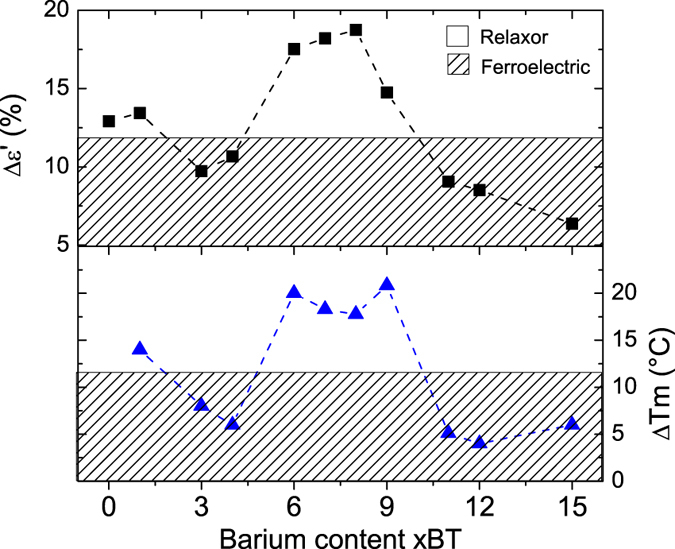
Quantification of frequency-dependent features in the dielectric permittivity curves of unpoled BNT-xBT samples. High values of Δε’ and ΔT_m_ are related to a relaxor state (see text). The hatched areas highlight compositions that exhibit a spontaneous ferroelectric state instead of a relaxor one.

**Figure 3:**
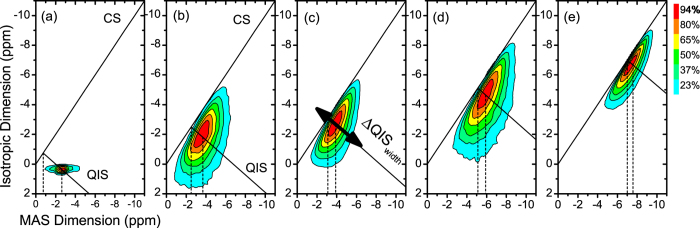
Contour plots of ^23^Na 3QMAS NMR spectra of **(a)** sodium acetate (a reference sodium salt) and representative BNT-xBT compositions: **(b)** BNT-0BT; **(c)** BNT-3BT; **(d)** BNT-6BT; and **(e)** BNT-12BT. The dashed lines represent the isotropic chemical shift (δ_ISO_) and the signal’s center of gravity (δ_CG_), respectively. The separation between both dashed lines corresponds to δ_QIS_. Diagonal solid lines represent the chemical shift (CS) and quadrupolar induced shift (QIS) lines. The fwhm along the QIS line corresponds to ΔQIS_width_, depicted by the double arrow in **(c)**. See Supporting Information for a detailed description of these parameters.

**Figure 4:**
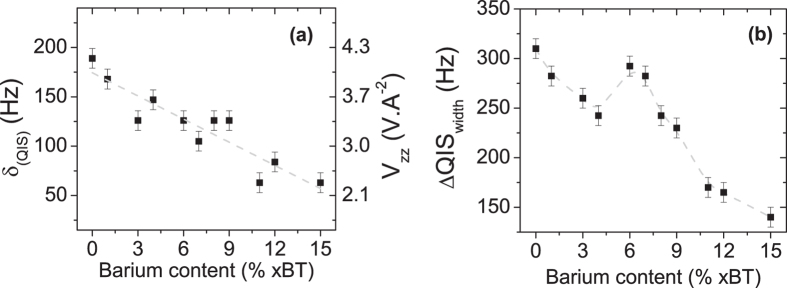
(**a**) Quadrupolar induced shift (left axes) and corresponding magnitude of mean EFG component (right axes). V_zz_ values are calculated with Eq. 1 and 2. **(b)** FWHM along the QIS line (ΔQIS_width_) as a function of barium content.

**Figure 5:**
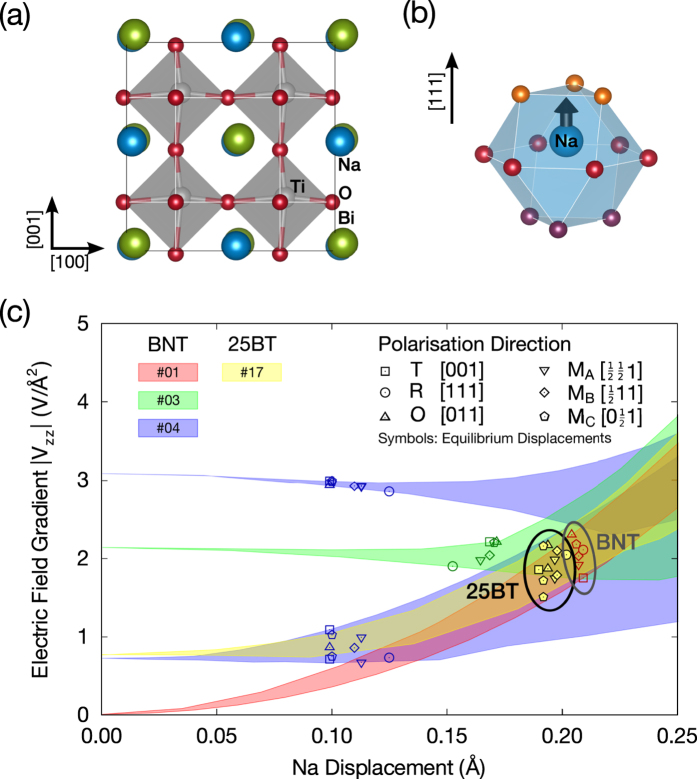
(**a**) polar displacements along [111] direction (space group R*3m*). **(b)** Distortion of sodium site due to ionic displacement. **(c)** Effect of polar distortions on calculated EFG values. Symbols depict the EFG for equilibrium values of sodium displacements for each polarization direction investigated. Shaded areas delimit the full range of |V_zz_| calculated for six polarization directions in a given chemical configuration. Four chemical configurations are considered (BNT: #01 rock-salt, #03 layers, #04 criss-cross lines; BNT-25BT: #17 rock-salt variant).

**Figure 6:**
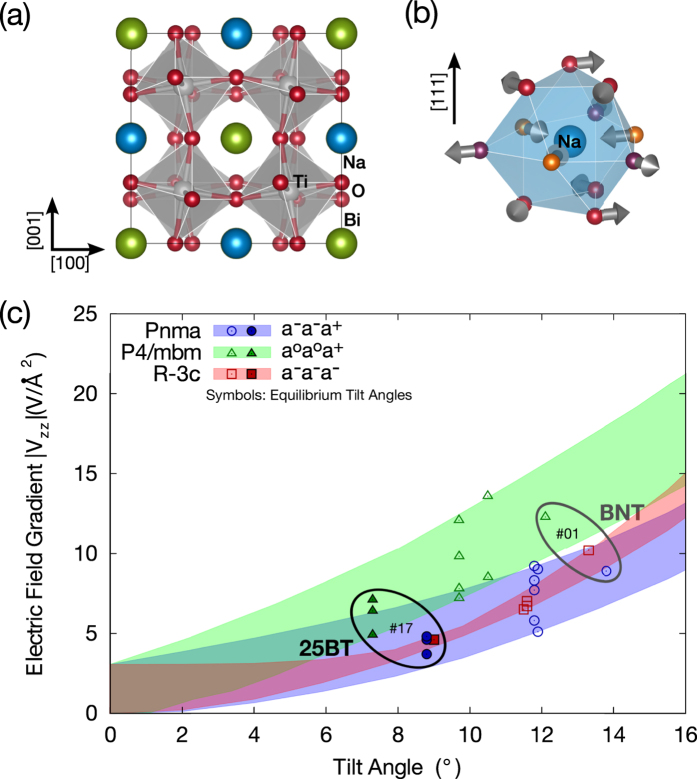
(**a**) crystal structure with *a*^−^*a*^−^*a*^−^ octahedral tilt (space group *R-3c*). **(b)** Distortion of sodium site due to octahedral tilting. Arrows indicate displacements of oxygen atoms. **(c)** Calculated EFG for different tilt systems. Symbols: EFG values of equilibrium tilt angles. Shaded areas: Full range for four different chemical configurations (same as Fig. 5).

The results section is structured as follows: In the first part, dielectric permittivity data of BNT-xBT is presented as a function of barium content, in order to distinguish which compositional ranges display a relaxor or spontaneous ferroelectric state (Figs 1 and 2). In the second part, the ^23^Na 3QMAS NMR spectra are presented (Fig. 3), together with the composition dependence of two relevant parameters, δ_QIS_ and ΔQIS_width_ (Fig. 4). These parameters are connected to the magnitude and the distribution of the EFG, respectively; the latter displays a strong correlation to the presence of a relaxor state in BNT-*x*BT. In the last part, DFT calculations exploring the impact of three distortion mechanisms on the EFG are presented (Figs 5 and 6), which demonstrate the predominant influence of octahedral tilting on the EFG.

### Spontaneous electric state of BNT-xBT

Figure 1 presents the temperature-dependent dielectric permittivity spectra of unpoled specimen of BNT-*x*BT. The displayed compositions were selected based on the similarity in the shape profile, such that BNT-0BT, BNT-3BT, BNT-6BT, and BNT-12BT represent the compositional ranges with barium content (in mole percent) of 0 ≤ x ≤ 2, 3 ≤ x ≤ 4, 5 ≤ x ≤ 10 and 11 ≤ x ≤ 15, respectively.

A marked feature can be observed in the permittivity curves of BNT-3BT and BNT-12BT below 200 °C. This frequency-independent event with sharp inflection point in ε’ was previously referred to as the depolarization temperature^32^. This temperature was later identified as *T*_F-R_, where a spontaneous ferroelectric order reverts to a relaxor state during heating^33^. The presence of such a profile indicates that samples BNT-3BT and BNT-12BT, as well as the composition ranges they represent, exhibit a spontaneous ferroelectric state at room temperature.

Contrastingly, only a broad component is present in the dielectric spectra of compositions which do not exhibit this sharp anomaly, namely BNT-0BT and BNT-6BT. This broad component features intense dispersion in ε’. In addition to that, the temperature of maximum ε” is frequency dependent, whereas it is invariant for compositions BNT-3BT and BNT-12BT. Such a dispersion of the permittivity has been attributed to the thermal relaxation of polar nanoregions both in lead-containing^34^ and BNT-based^5^ relaxor ferroelectrics. The presence of a dispersion in the permittivity data together with the absence of the sharp T_F-R_ transition indicate that the samples BNT-0BT and BNT-6BT are found in a relaxor state at room temperature. The same can be stated about the composition ranges these two samples represent.

The spontaneous relaxor or ferroelectric state of unpoled BNT-xBT solid-solutions is summarized in Fig. 2. Two parameters related to the dispersion of the dielectric permittivity are plotted as a function of barium content (xBT). The upper part of Fig. 2 depicts Δε’^6^, which is the relative decrease in ε’ between measuring frequencies of 1 kHz and 100 kHz, determined here at the T_F-R_ of each composition. The second parameter (ΔT_m_) is the frequency-induced shift of the temperature of the maximum ε” in the same frequency range. High values of Δε’ and ΔT_m_ correspond to a strong dispersion in the dielectric permittivity, and consequently quantify the relaxor state^34^. In contrast, the values found in the hatched area correspond to those compositions that exhibit both a pronounced T_F-R_ for unpoled samples and a suppressed dispersion of the permittivity and are thus in a spontaneous ferroelectric state.

A temperature shift ΔT_m_ of 14 °C is observed for BNT-1BT, which is an indicator of a large dispersion of the permittivity, and hence, a relaxor state. This parameter decreases to around 6 °C for BNT-3BT and BNT-4BT, indicating that features of a relaxor state are suppressed for barium contents close to 3%. Δε’ also decreases significantly in this composition range. The lower values of ΔT_m_ and Δε’ are indicative of a spontaneous ferroelectric state for BNT-3BT. Both ΔT_m_ and Δε’ rise for compositions around BNT-6BT, suggesting that a barium content higher than x = 6 disrupts the spontaneous ferroelectric order exhibited by BNT-3BT. Finally, for a BT content x = 11 and higher, the ΔT_m_ values decrease once more, indicating that frequency dispersion features are not very pronounced for compositions in this range. The suppressed frequency dispersion corroborates the fact that BNT-12BT and compositions with higher barium content exhibit a spontaneous ferroelectric order even prior to electric poling.

The composition-induced switch between relaxor and ferroelectric states in BNT-xBT, shown in Fig. 2, is quite unusual and cannot only be explained in terms of the average structure. Therefore, we employed ^23^Na NMR as a structure probe at the atomic scale to investigate to what extent the local structure of BNT-xBT influences its spontaneous electric state.

### ^23^Na 3QMAS Spectra of BNT-xBT

Figure 3(a) displays the contour plot of the ^23^Na 3QMAS NMR spectrum of a reference sodium salt (sodium acetate), which presents a signal with the shape of a narrow horizontal ridge. This signal is broadened only parallel to the MAS dimension, a shape characteristic of the quadrupolar coupling of 2^nd^ order for powder samples^35^. Furthermore, such a shape is characteristic of a material with a well-defined local structure.

This picture contrasts strongly to the ^23^Na 3QMAS NMR spectra of BNT-xBT materials. Figure 3(b–e) exhibits spectra of the same compositions displayed in Fig. 1. All spectra display a broad signal that is broadened both in the MAS and in the isotropic dimensions, besides being stretched along both the CS and QIS lines. These marked differences in the signal shape between a reference sodium salt and BNT-xBT suggest that the ceramic samples exhibit a significant degree of disorder in their local structure. This disorder can be quantified in terms of the quadrupolar interaction and the parameters used to describe it (a detailed description of the interpretation of 3QMAS NMR spectra is provided in the Supporting Information).

The quadrupolar induced shift (δ_QIS_) is a spectral parameter related to the magnitude of the EFG^35^. The size of δ_QIS_ is equal to the separation between both vertical dashed lines displayed in each spectra of Fig. 3. These lines correspond to the isotropic chemical shift (δ_ISO_) and the signal’s center of gravity δ_CG_, respectively. A comparison between the spectra of different compositions indicates δ_QIS_ varies with the barium content, as both BNT-6BT and BNT-12BT exhibit smaller values than pure BNT. This trend is also observed when δ_QIS_ is plotted as a function of BT content (xBT) in Fig. 4a. Although the values are rather scattered, a monotonic decrease of δ_QIS_ occurs as the barium content increases. Since δ_QIS_ is directly related to the EFG, its decrease corresponds to a decrease in the magnitude of EFG main component (V_zz_) as barium is added to the structure.

The intensity of this effect can be illustrated by comparing both ends of the investigated series individually. While pure BNT exhibits a δ_QIS_ of 189 Hz, this parameter amounts to 63 Hz for BNT-15BT. Taking into account the quadratic relation between δ_QIS_ and the EFG^35^ and assuming an asymmetry parameter of zero for this tensor, these values of δ_QIS_ can be translated into an EFG decrease of approximately 40%, with a V_zz_ of 4.2 and 2.4 VÅ^−2^ for BNT-0BT and BNT-15BT, respectively. A decrease of 20% in V_zz_ is observed between pure BNT and BNT-6BT as well.

Although the relation between δ_QIS_ and barium content exposed in Fig. 4a already enables a preliminary characterization of the quadrupolar coupling in the BNT-xBT system, this is only one aspect of the local structure in these ceramics. It is shown above that the ^23^Na 3QMAS NMR signals are continuously broadened along the QIS line and, therefore, exhibit features of a distribution of NMR parameters (Fig. 3(b–e)). Hence, the system cannot be characterized by a single value of δ_QIS_; instead, it is better described by a distribution thereof, which mean value is related to the δ_QIS_ parameter displayed in Fig. 4a.

In light of this fact, a further aspect of interest is how this distribution evolves as a function of barium content. Here, we propose a straightforward approach for estimating the EFG distribution width and, consequently, the degree of disorder in the local structure. If a distribution of δ_QIS_, and therefore EFG values, is present, it should result in a signal stretched along the QIS line for a given isotropic chemical shift, due to the quadrupolar induced shift. Therefore, the broader the distribution of EFG values, the broader the signals will be in this specific direction. Hence, the full width at half maximum (fwhm) along the QIS line (ΔQIS_width_) is a measure for the variety of local environments around the sodium nuclei (see Supporting Information for additional details).

All BNT-xBT compositions investigated exhibit a ^23^Na 3QMAS signal considerably broadened along the QIS line, regardless of their barium content. In addition to that, the signal width along this direction changes markedly as a function of composition (Fig. 3). While a broad signal is observed for samples with low barium content, much narrower lines are observed for BNT-12BT. Moreover, a close inspection of ΔQIS_width_ reveals a narrower profile for BNT-3BT when compared to other compositions with low and intermediate barium content (e.g., BNT-0BT and BNT-6BT).

This analysis is extended to the complete series of compositions studied. Figure 4b displays the values of ΔQIS_width_ plotted as a function of barium content. Pure BNT exhibits a width of 320 Hz, indicating a large variety of local environments. With a small addition of barium (e.g. BNT-3BT) this width drops approximately 20% to a value of 250 Hz. This narrower line could indicate a less disordered local structure, with a narrower distribution of local environments. A further addition of barium, for example at BNT-6BT, results in an increase of ΔQIS_width_ to values close to that of pure BNT. Further increase of the barium content (e.g. BNT-12BT or BNT-15BT) reduces ΔQIS_width_ to 50% of that exhibited by BNT-0BT.

This trend presents strong similarities to the behavior of ΔT_m_ as a function of barium content displayed in Fig. 2, suggesting a correlation between ΔQIS_width_ (a measure of the distribution of EFG) and features of the relaxor state in a given BNT-xBT composition. This fact raises the following question: what structural motifs contribute most to the EFG value around sodium nuclei? The answer might bring us a step closer to understanding the relation between the spontaneous electric state displayed by BNT-xBT samples and their local structure.

### DFT Calculations of Electric Field Gradients (EFG)

The ideal perovskite structure provides cubic point symmetry at the A-site and, hence, an EFG equal to zero at the position of the sodium nuclei. Any structural distortion will result in an EFG ≠ 0 at the sodium position with the value of the EFG being proportional to the distortion’s magnitude. In the following, we investigate how the three fundamental mechanisms of symmetry breaking in these perovskites, namely cation arrangement, ionic displacements (Fig. 5a,b) and octahedral tilting (Fig. 6a,b), contribute to the electric field gradient’s main component (|*V*_zz_|) on the sodium site. Furthermore, we are interested in analyzing the influence that the addition of barium has on each distortion mechanism analyzed, in order to identify the local structural feature which is responsible for the pronounced decrease of EFG values as a function of increasing barium content.

For this purpose, the EFG on the sodium sites of several structure models was calculated using density function theory (DFT). Calculations were performed for models of pure BNT as well as barium-containing ones, taking different chemical configurations (i.e. the arrangement of neighboring A-site cations) into account. Using similar calculation parameters as in this study, the simulation analogue of the morphotropic composition of the BNT-*x*BT was found at about x = 35%^36^. Therefore, we choose the compositions BNT and BNT-25BT for the DFT calculations as representatives of pure BNT and BNT-*x*BT on the rhombohedral side of the MPB (i.e. *x* ≈ 5%).

### Cation Arrangement

EFG values are calculated for various chemical configurations to consider the effect of random A-site occupancy in a simplified way. Twelve chemical configurations are explored in total (Figure S2), six of which contain two barium atoms substituting one bismuth and one sodium cation. The atomic positions were structurally relaxed prior to determination of |*V*_zz_|. This procedure allows us to access the range of EFG values that should be present in the real disordered materials.

Contrasting to the assumption that a third cation type in the next-nearest neighbor A-site sphere of sodium would lead to a different range of EFG values on this site, the addition of barium did not result in any significant change in the range of calculated EFG values. For pure BNT configurations, |V_zz_| values lie between 0 and 3 V/Å^2^ (Figure S3) and exhibit hardly any dependence on the unit cell volume. Barium-containing chemical configurations display a similar range of |V_zz_| values to those calculated for pure BNT. The rock-salt arrangement (#01), in which all sodium ions are surrounded exclusively by bismuth ions, exhibits an EFG equal to zero when no other distortion mechanism is present. These results demonstrate that the A-site cation arrangement indeed influences the EFG on the sodium site. However, its effect differs from the experimentally observed trend of decreasing EFG upon addition of barium. Therefore, a different symmetry breaking mechanism should account for this experimental finding.

### Polarization Direction and Amplitude

The effect of polarization amplitude was investigated with respect to polarization direction. Variable amplitudes of the polar vector were considered, for which all atoms are shifted along a specific direction. Figure 5c displays the dependence of |V_zz_| as a function of sodium displacement in each of these cases. Shaded areas represent the full range of EFG values for all six polarization directions considered. Four chemical configurations are analyzed, three of pure BNT and one of BNT-25BT (models #01, #03, #04 and #17, respectively), which are illustrated by different colors. Configuration #04 bears two non-equivalent sodium sites, depicted by the two blue shaded areas.

At lower polar displacements (up to 0.05 Å) the shaded areas are narrow and rather independent of the amplitude and direction of polar displacement, suggesting the EFG imposed by the cation arrangement is predominant. These areas broaden slightly between 0.10 and 0.20 Å. This range contains the equilibrium values of polar displacement (i.e. the displacement with minimal total energy), which are depicted as open symbols in Fig. 5c. EFG values found at equilibrium displacements span over less than 1 V/Å^2^ for different polarization directions (e.g. [111] or [100]) in a given chemical configuration. This span is much smaller than that observed for the influence of cation arrangements alone. This result suggests that the main component of the EFG (|V_zz_|) on sodium nuclei depends only weakly on the direction of polar displacement in these perovskites.

Despite this fact, there is a clear dependence between |V_zz_| and the magnitude of sodium displacements for the rock-salt configuration (#01), with increasing |V_zz_| for larger displacements (Fig. 5c - red shaded area). The effect of the A site occupancy can be disregarded when this specific chemical configuration is considered, because its next-nearest neighbor arrangement does not contribute to the EFG. Hence, this dependence indicates that the polarization amplitude influences the calculated values of the EFG.

At this point one should evaluate how the polar cation displacements are affected by the addition of barium to the models investigated. The role of barium addition can be analyzed by comparing the BNT-25BT model #17 to the pure BNT model with rock-salt configuration (#01). Although the barium-containing chemical configuration exhibits a |V_zz_| close to 1 V/Å^2^ before any polar displacement is imposed (a consequence of its cation arrangement), its progressions with increasing polar displacement is very similar to that of pure BNT (#01). Furthermore, the ranges of |V_zz_| for the equilibrium values of displacement lie very close for both configurations. These observations indicate that the addition of barium has little effect on the magnitude of cation displacements and the resulting EFG value. Since the trend of EFG values calculated for polar displacements does not reflect the one experimentally observed, another distortion mechanism must be predominantly responsible for the EFG on the sodium site.

### Octahedral Tilt

The third structural distortion mechanism that may contribute to the EFG at the sodium site is the deformation of the oxygen cuboctahedron that occurs as a consequence of octahedral tilting (Fig. 6a,b). EFG values were calculated for three different tilt systems as a function of octahedral tilt amplitude (i.e. different tilt angles) in the absence of cation displacements. Figure 6c displays |V_zz_| values calculated for three tilt systems that are relevant for the structure characterization of BNT (*a*^−^*a*^−^*a*^−^, *a*^−^*a*^−^*a*^+^ and *a*^*o*^*a*^*o*^*c*^+^). Shaded areas in Fig. 6c indicate the full range of EFG values found for a given tilt system when analyzed for the same four chemical configurations considered in Fig. 5. While symbols of the same color as the shaded area indicate the equilibrium values of the tilt angles for pure BNT models, filled symbols stand for the barium containing model. These EFG values correspond to the minimum value of total energy and are, hence, relevant for the real crystal structure. Symbols for the rock-salt model of BNT (#01) and its barium-containing variant are circled for better visualization.

|V_zz_| values resulting from octahedral tilting lie between 5 and 13.5 V/Å^2^ at relevant tilt angles and, therefore, are much higher than those resulting from cation arrangement or polarization (0 to 3 V/Å^2^). Besides that, the most important fact we can learn from the calculations is that a significant decrease of |V_zz_| occurs between pure BNT and barium containing models for all three tilt systems investigated. While the range for *a*^−^*a*^−^*a*^−^ (squares) decreases from between 6.5 and 10 V/Å^2^ to about 4.5 V/Å^2^ upon addition of barium, the range for *a*^−^*a*^−^*a*^+^ (circles) decreases from (5–9) to (4–5) V/Å^2^. Additionally, the range for *a*^*0*^*a*^*0*^*a*^+^ (triangles) decreases from (7–13.5) to (5–7) V/Å^2^ when barium is added.

The equilibrium values of tilt angles are also considerably smaller when barium is present, demonstrating the tilt suppression effect of this cation. While the theoretical tilt angles for pure BNT are in the range of 11.5–14° in *R-3c* and *Pnma* models and 10–12° in the *P4/mbm* one, they decrease to between 7° and 9° for the barium containing model, which is the simulation analogue of rhombohedral compositions near the MPB (e.g. BNT~5BT). The equilibrium values of tilt angle calculated here are larger than the experimental ones (e.g. 8° in the *R3c* structure of BNT^7^). Nonetheless, this fact is not relevant considering the aim of these calculations, which is to determine the qualitative effect of the presence of barium in the structural models investigated. With this respect, the DFT calculations presented here demonstrate that the addition of barium results in a decrease of the calculated EFG values by about 50%, regardless of the tilt system considered. From this observation, we may conclude that octahedral tilting is the only mechanism of structural distortion able to reproduce the experimental finding of halved mean EFG values upon addition of barium to BNT.

## Discussion

Based on the results of our DFT calculations, we can infer that the experimental EFG on the sodium site reflects the octahedral tilting in BNT-xBT. Tilting of oxygen octahedra has a high impact on the calculated EFG values, which is much higher than that of A-site occupancy or polar distortions alone. Furthermore, the DFT calculations reproduce the experimental trend of a marked decrease of EFG values upon the addition of barium only when tilting is considered. The larger ionic radius of Ba^2+^ (1.61 Å)^37^, when compared to those of Bi^3+^ (1.45 Å) or Na^+^ (1.39 Å), can be considered responsible for the suppression of octahedral tilt in BNT-*x*BT^11^,^36^ and other bismuth containing perovskites (e.g. BiFeO_3_-xBT)^38^; thus, further supporting the relation between octahedral tilting and the experimental EFG at the sodium site together with their decrease whenever barium is added to the structure.

The complexity of the structure of BNT and its barium doped versions has been often described by the coexistence of two different tilt systems. As an example, the coexistence between *a*^−^*a*^−^*a*^−^ and *a*^−^*a*^−^*c*^+^ tilt systems has been reported for pure BNT^9^, and the coexistence of *a*^−^*a*^−^*a*^−^ and *a*^*0*^*a*^*0*^*c*^+^ tilt systems has been reported for MPB compositions (e.g. BNT-6BT)^5^. The coexistence of two different tilt systems should result in two distinct NMR signals, each one displaying a sharp and well defined powder pattern, similar to the reference sodium salt (Fig. 3a). However, the ^23^Na NMR spectra of BNT-xBT materials display a unimodal distribution of EFG values instead. This fact implies that the local structure of BNT-xBT ceramics is better described by a unimodal distribution of octahedral tilting instead of the coexistence of two in-phase and out-of-phase tilt systems, as far as the point of view of NMR is concerned.

This picture of the local structure may contrast to the average structure determined with diffraction methods. Nonetheless, it supports recent contributions that evoked a disordered local environment to explain the structure and properties of BNT-based materials. Levin and Reaney^16^ recently reported that the coexistence of localized in-phase and long-range anti-phase tilts in pure BNT may be accommodated by continuously varying tilting magnitude and direction, in order to alleviate the strain of boundaries between regions with different tilt orientation. Furthermore, Rao and Ranjan^9^ proposed that the structural and dielectric properties of BNT are a consequence of strain heterogeneities caused by the localized in-phase regions, which are incompatible with the *a*^−^*a*^−^*a*^−^ “matrix”. The importance of the competition between two tilt systems has also been highlighted by Garg and Ranjan for the nature of structure and polar states in BNT-6BT^39^. The results from NMR further support these views of the structure of BNT and its tilt disorder.

Furthermore, it is observed that the extent of local structure disorder is a function of the barium content (Fig. 4b). Based on the relation between the EFG on sodium sites and the octahedral tilting, the width of the EFG distribution (ΔQIS_width_) should represent the degree of tilt disorder in the structure. Interestingly, a comparison between the composition dependence of the dispersion of dielectric permittivity (ΔT_m_ – Fig. 2) and that width of the EFG distribution (ΔQIS_width_ – Fig. 4b) shows that they both display the same barium content dependence. Compositions found in a relaxor state exhibit higher values of ΔQIS_width_ (e.g., BNT-0BT and BNT-6BT) than those which display a spontaneous ferroelectric ordering (e.g., BNT-3BT and BNT-12BT). Based on this correlation and since ΔQIS_width_ is a measure of the distribution of octahedral tilting, we propose that substantial disorder in octahedral tilting is a characteristic feature of the relaxor state in BNT-xBT materials.

From the wider distribution of EFG, on the local scale, and the apparent coexistence of two tilt systems, on a macroscopic scale, one could assume the occurrence of a relaxor state in pure BNT and BNT-6BT is a consequence of tilt disorder. However, it is sounder to interpret the correlation between tilt disorder and the relaxor behavior in BNT-xBT in the framework of both features having a common source. This common source might be the strain caused by the competition between different structures in the system, a characteristic possibly related to the random A-site occupancy in these perovskites.

In the scenario of continuous octahedral tilting disorder, the cubic phase recently revealed by NMR experiments^13^ could be regarded as a smooth interface between regions of more marked tilting or regions with different tilt orientation^40^. Although this cubic content cannot be detected with 3QMAS, as this experiment is “blind” for sites with vanishingly small EFGs, a larger content of the cubic phase is reported^13^ for samples with higher degree of local disorder (e.g. the ones characterized in a relaxor state).

## Summary and Conclusion

In summary, we performed a complementary study between NMR, dielectric relaxation and DFT to reveal the salient role that local structural features play for the irregular occurrence of relaxor behavior in the lead-free BNT-xBT series. From two-dimensional ^23^Na 3QMAS NMR experiments, the electric field gradient (EFG) on the sodium site is elucidated. Its analysis reveals a distribution of local structural motifs, quantified by the width (ΔQIS_width_) of the EFG distribution. ΔQIS_width_ exhibits a peculiar composition dependence, which closely mimics the intensity of frequency dispersion in dielectric spectra and is reported for the first time. A broader distribution of EFG values is observed for compositions found in a relaxor state than the ferroelectric state. Based on DFT calculations, we can infer that the experimental EFG on sodium site reflects the behavior of octahedral tilting in BNT-xBT.

Furthermore, the correlation found between ΔQIS_width_ and the dispersion of dielectric permittivity strongly indicates that octahedral tilting disorder is a local structural feature concurrent with the occurrence of relaxor behavior in these lead-free electrically functional ceramics. Finally our results solve the question about the distribution of tilt-angles in these materials. From our results we can rule out the local coexistence of two well defined tilt systems and corroborate the continuous distribution of tilting angle and tilt direction model proposed by Levin & Reaney^16^.

## Methods

### Materials

A conventional solid state reaction route was applied to prepare powders with compositions of (100-*x*)(Bi_1/2_Na_1/2_)TiO_3_ + *x*BaTiO_3_ (0 ≤ *x* ≤ 15), referred to as BNT-*x*BT hereafter. Powders of Bi_2_O_3_, Na_2_CO_3_, TiO_2_, and BaCO_3_ were used as raw materials. These were weighed conforming to the intended stoichiometry and ball-milled for 24 h in anhydrous ethanol with zirconia balls. Subsequent drying and calcination at 800 °C for 3 h were followed by a cold-isostatic press at 300 MPa. Resulting discs were sintered in a covered alumina crucible at 1150 °C for 3 h under the atmosphere of the same composition.

### Dielectric spectra

Electrical measurements were carried out after applying Ag paste on both sides of a disk-shaped specimen and subsequent firing at 700 °C for 30 min. The temperature-dependent dielectric permittivity of the samples was recorded with an impedance analyzer (HP4284A) at a heating rate of 2 K/min.

### Nuclear Magnetic resonance

Two dimensional ^23^Na 3QMAS (or MQMAS) NMR spectra were acquired with a CMX 600 VARIAN Infinity+ spectrometer with a carrier frequency of 158.751800 MHz. A Z-filter pulse sequence was employed^41^, with excitation, conversion and Z-filter pulse durations of 4.25 μs, 1.65 μs and 24.5 μs, respectively. The Z-filter delay was 2 ms long, and 128 t1 increments of 10 μs were recorded, with a recycle delay of 2.0 s, and 240 scans for each increment. Ceramic pellets of BNT-xBT were crushed to powders and annealed at 400 °C for 2 h. This condition is reported as sufficient high to relieve mechanical stresses caused by grinding^42^. The resulting powders were spun under MAS conditions at 10 kHz in 5 mm zirconia rotors. All spectra presented were sheared to achieve the pure CS- and QIS-axes. The chemical shift scale was referenced to a 1 M NaCl aqueous solution (0 ppm). A Matlab script was developed to extract slices from the 2D NMR spectra, determine the width along the QIS for the maximum of the signals (ΔQIS_width_) as well as the quadrupolar shift (δ_QIS_), determined as the distance from the signal’s center of gravity (δ_CG_) to the isotropic line.

### DFT calculations

Two specific materials were chosen for the computation with the reason for this particular choice rationalized in the results section. Electric field gradient tensors at ^23^Na in Bi_1/2_Na_1/2_TiO_3_ and 75Bi_1/2_Na_1/2_TiO_3_ -25BaTiO_3_ were obtained using the Vienna ab-initio simulation package (VASP, version 5.3)^43^,^44^,^45^,^46^. Projector augmented plane waves (PAW)^47^,^48^ were applied with the local density approximation (LDA) exchange correlation functional^49^. As the calculation of electric field gradients requires very high accuracy, therefore semicore GW-potentials (Na 3*s*^2^ 3*p*^6^ 4*s*^1^, Bi 5*d*^10^ 4*s*^2^ 4*p*^3^, Ba 5*s*^2^ 5*p*^6^ 6*s*^2^, Ti 3*s*^2^ 3*p*^6^ 4*s*^2^ 3*d*^2^, O 2*s*^2^ 2*p*^4^) were used with a kinetic cutoff energy of 800 eV. The Monkhorst-Pack *k*-point^50^ mesh for Brillouin zone integration was set to 8 × 8 × 8 with respect to the perovskite primitive cell resulting in overall errors of less than 0.05 V/Å^2^ for EFGs and 0.1 meV/f.u. for total energies.

## Additional Information

**How to cite this article**: Groszewicz, P. B. *et al*. Reconciling Local Structure Disorder and the Relaxor State in (Bi_1/2_Na_1/2_)TiO_3_-BaTiO_3_. *Sci. Rep*. **6**, 31739; doi: 10.1038/srep31739 (2016).

## Supplementary Material

Supplementary Information
