# Supplementary material for: Reconciling Local Structure Disorder and the Relaxor State in (Bi1/2Na1/2)TiO3-BaTiO3
Source: Sci Rep. 2016 Aug 22;6:31739. doi: 10.1038/srep31739 (PMC4992844; doi:10.1038/srep31739)
Supplement: Supplementary Information [file srep31739-s1.pdf]

Supporting Information

Reconciling Local Structure Disorder and the Relaxor State in $(Bi_{1/2}Na_{1/2})TiO_3$-$BaTiO_3$

Pedro B. Groszewicz[1], Melanie Gröting[2], Hergen Breitzke[1], Wook Jo[3], Karsten Albe[2], Gerd Buntkowsky[1] and Jürgen Rödel[2*]

[1]Institute of Physical Chemistry, Technische Universität Darmstadt, 64287 Darmstadt, Germany
email: gerd.buntkowsky@chemie.tu-darmstadt.de
[2]Institute of Materials Science, Technische Universität Darmstadt, 64287 Darmstadt, Germany
email: roedel@ceramics.tu-darmstadt.de
[3]School of Materials Science and Engineering, Ulsan National Institute of Science and Technology, 689-798 Ulsan, Republic of Korea

ESI1. Interpretation of ^{23}Na NMR 3QMAS spectra

3QMAS NMR spectra provide two pieces of information, represented by the diagonal lines in Figure 1ESI. The first of these is the chemical shift (CS), a diagonal line with slope equal to 1, along which the chemical shift interaction is present. The second is the quadrupolar induced shift (QIS Line – displayed in green), which reveals the effects of the second order quadrupolar interaction on the apparent chemical shift. This interaction results in a shift of the signal's center of gravity away from the CS line. This shift occurs along the quadrupolar induced shift axis (QIS), a line with the slope of -10/17 in a sheared two-dimensional spectrum.[1] The farther the signal is located below the CS line, the larger the corresponding EFG at the nuclear site will be.

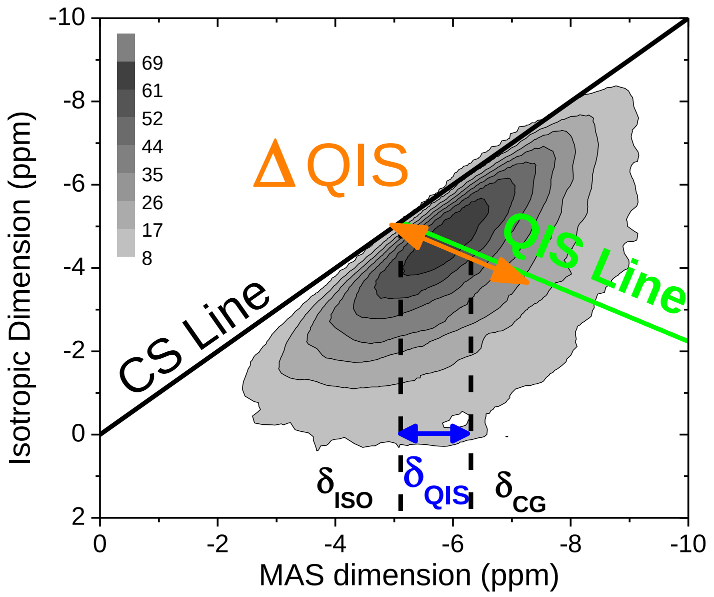

Figure 1ESI: Example of a ^{23}Na 3QMAS spectrum of BNT-xBT materials highlighting its relevant features concerning the quadrupolar coupling (δ_{QIS} and ΔQIS_{width}).

The magnitude of the components of the EFG tensor for ^{23}Na (a nucleus with a I=3/2 nuclear spin), can be determined from the magnitude of δ_{QIS},(in ppm) as described by equation 1,[2] where ν_L stands for the Larmor frequency, η for the asymmetry parameter of the EFG and C_Q for the quadrupolar coupling constant:

Eq 1. $$\delta_{QIS} = -\frac{1}{40}\frac{10^6}{\nu_L^2}\left(C_Q\sqrt{1+\frac{\eta^2}{3}}\right)^2$$

C_Q is a direct measure of the main component of the EFG (V_{zz}), as expressed by Eq. 2, where e stands for the elementary charge, Q for the quadrupolar moment of the nucleus and h for Planck's constant.

Eq. 2. $$C_Q = \frac{eQ}{h}V_{zz}$$

The quadrupolar induced shift δ_{QIS} is computed on the MAS dimension as the distance between the center of gravity of the signal (δ_{CG}) and the isotropic value of the chemical shift (δ_{Iso}) (see Eq. 3).

Eq. 3 $$\delta_{QIS} = \delta_{CG} - \delta_{ISO}$$

The δ_{ISO} can be determined as the point where the CS axis is intersected by the QIS line that passes through the signal's center of gravity, with its value read on the MAS dimension. Both δ_{ISO} and δ_{CG} are highlighted in Figure 1ESI as vertical dashed lines. Their separation amounts to δ_{QIS}, which is displayed by the blue double arrow line.

A further feature of the ^{23}Na 3QMAS NMR spectra of BNT-xBT concerns the signal width along the QIS line (ΔQIS_{width}). This parameter is obtained by measuring the full width at half height (fwhm) along the QIS line and is depicted as a orange double arrow in Figure 1 ESI. In the spectra of materials with a well-defined local structure, this width amounts to less than a half ppm (approximately 70 Hz under the experimental conditions reported here) and is therefore negligible. Contrasting to that, the signal width along the QIS line is very pronounced in spectra of BNT-xBT samples. Based on the overall signal's shape, and especially along the QIS line, this width (ΔQIS_{width}) can be considered as a measure of the distribution of quadrupolar coupling constants (C_Q), and hence the distribution of the magnitude of the main component of the EFG tensor (V_{zz}).

ESI2. Chemical configurations employed in DFT calculations

Thirteen different arrangements of the A-site cations have been considered in the DFT calculations reported in this work. They are displayed in Figure 2ESI below.

Figure 3ESI below presents the EFG values calculated for each of the thirteen chemical configurations displayed in figure 2ESI. Values calculated for chemical configurations based in pure BNT are displayed in blue, whereas those from barium containing models are displayed in red. No significant differences can be observed in the range of calculated values of models with or without barium, as both cases result in V_{zz} values ranging from 0 to 3 V/A^2.

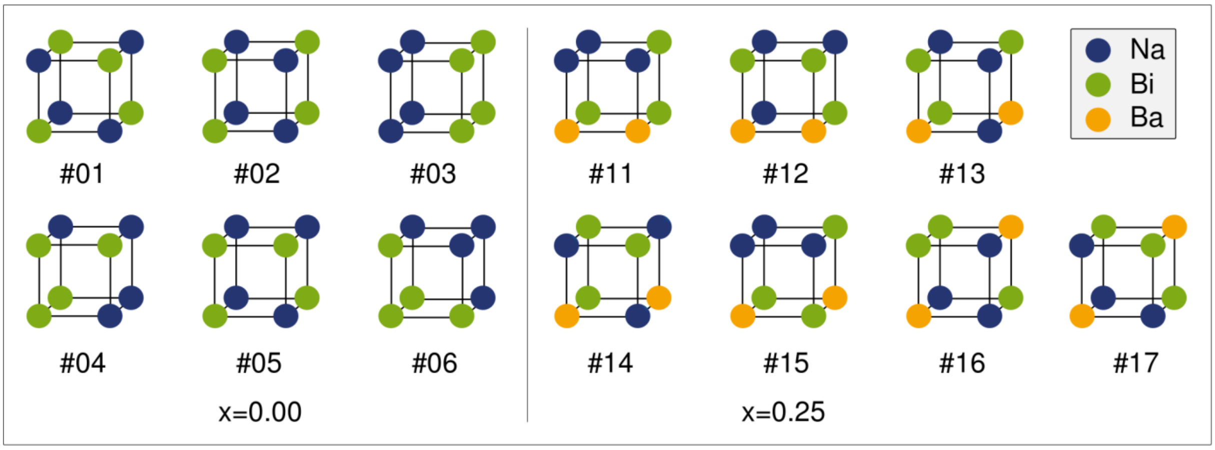

Figure 2ESI: Chemical configurations for BNT and BNT-25BT models

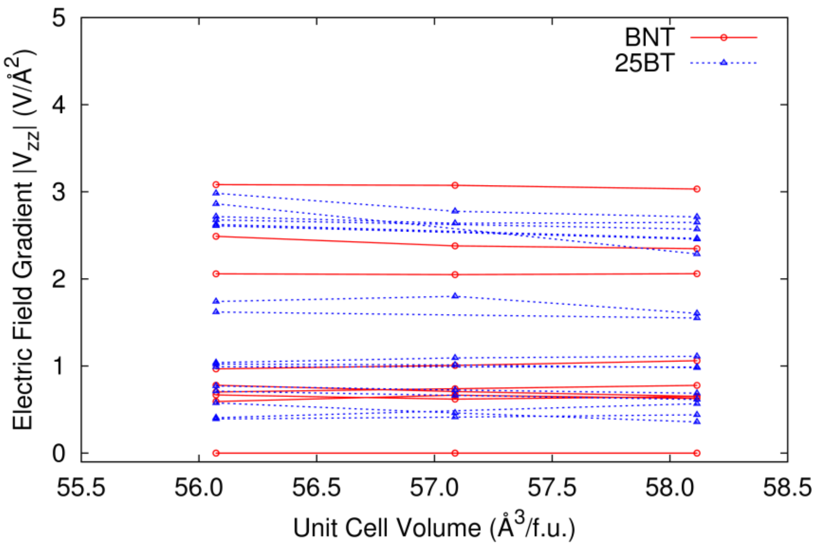

Figure 3ESI: Electric field gradients calculated for different chemical environments in models of BNT-0BT and BNT-25BT.

ESI3. Single pulse excitation ^{23}Na NMR spectrum of BNT-BT.

The magnitude and the presence of a distribution of the EFG can be evaluated both from the central transition of ^{23}Na in 3QMAS spectra or from its satellite transitions in one-dimensional spectra recorded with single pulse excitation. Figure 4ESI compares the ^{23}Na NMR spectrum of BNT-6BT to that of $NaNO_2$, where only the spinning sidebands of the satellite transitions are shown for clarity. The usual shape for a powder sample with a well defined EFG tensor is observed for $NaNO_2$, with clear maxima and singularities. Contrastingly, the BNT-6BT sample exhibits only a featureless Gaussian shaped envelope of spinning sidebands, what indicates a distribution of EFG components and, hence, a distribution of local environments for sodium in this material.

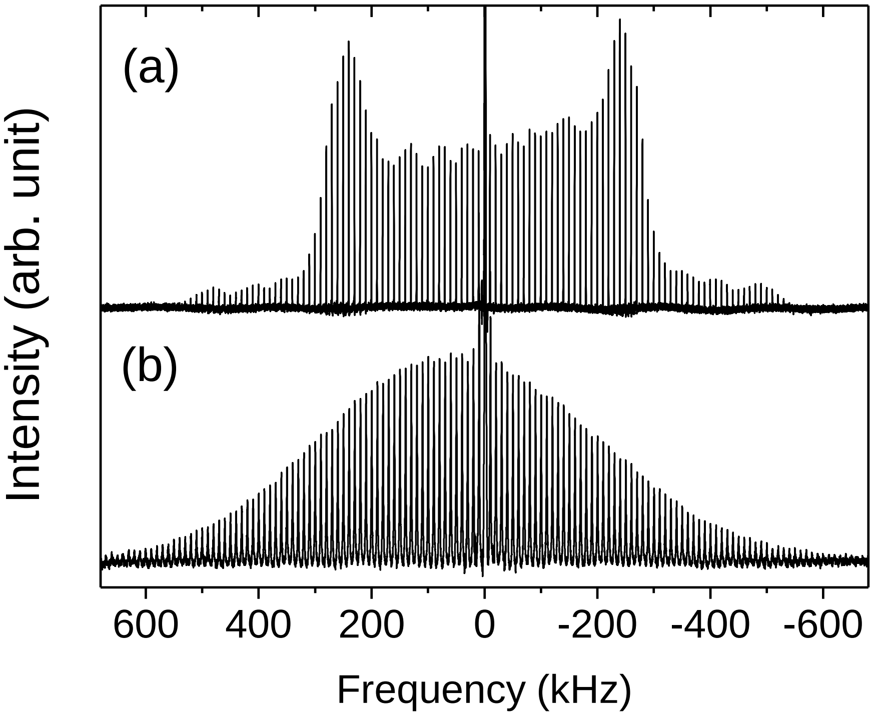

Figure 4ESI: One-dimensional single pulse ^{23}Na NMR spectrum of (a) $NaNO_2$ (for comparison of a material with a unique, well-defined EFG) and (b) BNT-6BT, recorded under magic angle spinning at 10 kHz in a 14.1T magnet. Only the satellite transitions are presented in order to highlight the shape caused by a single EFG tensor (a) and the distribution of EFGs (b).

Bibliography

[1] J.-P. Amoureux, M. Pruski, in *Encyclopedia of Magnetic Resonance*, John Wiley & Sons, Ltd, 2007.
[2] J. P. Amoureux, C. Fernandez, Solid State Nuclear Magnetic Resonance 1998, 10, 211.
